# Efficient Oil/Water Separation Membrane Derived from Super-Flexible and Superhydrophilic Core–Shell Organic/Inorganic Nanofibrous Architectures

**DOI:** 10.3390/polym11060974

**Published:** 2019-06-03

**Authors:** Zhi Liu, Detao Qin, Jianghui Zhao, Quan Feng, Zhengtao Li, Hongwei Bai, Darren Delai Sun

**Affiliations:** 1 School of Textile and Garment, Anhui Polytechnic University, Beijing Road, Wuhu 241000, China; zhaojh@ahpu.edu.cn (J.Z.); fengquan@ahpu.edu.cn (Q.F.); 2 Energy Research Institute @ NTU, Interdisciplinary Graduate School, Nanyang Technological University, Singapore 639798, Singapore; dqin001@e.ntu.edu.sg; 3 School of Civil and Environmental Engineering, Nanyang Technological University, Singapore 639798, Singapore; ZLI018@e.ntu.edu.sg

**Keywords:** core shell, flexible, oil water separation, superhydrophilicity, underwater superoleophobicity

## Abstract

To address the worldwide oil and water separation issue, a novel approach was inspired by natural phenomena to synthesize superhydrophilic and underwater superoleophobic organic/inorganic nanofibrous membranes via a scale up fabrication approach. The synthesized membranes possess a delicate organic core of PVDF-HFP and an inorganic shell of a CuO nanosheet structure, which endows super-flexible properties owing to the merits of PVDF-HFP backbones, and superhydrophilic functions contributed by the extremely rough surface of a CuO nanosheet anchored on flexible PVDF-HFP. Such an organic core and inorganic shell architecture not only functionalizes membrane performance in terms of antifouling, high flux, and low energy consumption, but also extends the lifespan by enhancing its mechanical strength and alkaline resistance to broaden its applicability. The resultant membrane exhibits good oil/water separation efficiency higher than 99.7%, as well as excellent anti-fouling properties for various oil/water mixtures. Considering the intrinsic structural innovation and its integrated advantages, this core–shell nanofibrous membrane is believed to be promising for oil/water separation, and this facile approach is also easy for scaled up manufacturing of functional organic/inorganic nanofibrous membranes with insightful benefits for industrial wastewater treatment, sensors, energy production, and many other related areas.

## 1. Introduction

Membrane separation is a popularly used technology for wastewater treatment, water purification, seawater desalination, and even oil/water separation [1,2,3,4,5]. Since its first development in the 1960s, polymer-based membranes have dominated the market in various applications field from the pharmaceutical/medical industry, food processing, gas separation, water purification and treatment, electronic devices, etc., with a huge number of membranes emerging in the market [6,7,8].

However, conventional polymer-based membranes synthesized by phase inversion processes not only involve a complicated process with lots of chemical consumption and waste gas/water generation, but also have a rough and hydrophobic surface, which is easy to be fouled by oil or other matter [9]. Membrane fouling owing to the accumulation of pollutants in the pore or on the surface of the membrane has become a bottleneck, limiting future membrane applications by painting it with an image of shorter lifespan, poorer water quality, lower water productivity, and higher energy consumption, which is contradictory to a sustainable and green development future [10,11].

An ideal separation membrane is featured with special properties such as flexibility for ease of application, superhydrophilicity for low fouling tendency and high productivity, and good mechanical strength for a longer lifespan. New material development with interdisciplinary innovation creates a lot of opportunities to make a breakthrough by integrating the advantages of well-designed next generation separation membranes [6] and continuous spinning technology [12]. To the best of our knowledge, few reports were published about the integration of organic backbone structures and inorganic surface properties with outstanding attributes and performances to address the severe oil/water separation issue.

Nature is a good school [13] and inspires us a lot to create many wonderful materials with promising applications to address practical engineering problems. In the past decades, special wettability membranes have been designed in combination with surface roughness and chemistry [14]. Jiang et al. firstly reported a superhydrophobic PTFE coated mesh film for oil/water separation [15]. Since then, membranes with superhydrophobicity have been intensively studied and applied to separate oils from water [16,17,18]. However, as water is denser than oil, it tends to form a barrier between the oil and hydrophobic membranes, preventing oil permeation through the membrane, and limiting the performance of oil and water separation to great extent. In addition, hydrophobic membranes always have an affinity to the oil phase, causing a high tendency of membrane fouling and clogging [19].

On the contrary, hydrophilic membranes, such as cellulose acetate membrane (HTI company) or emerging ceramic membranes, exhibit a low fouling tendency, while cellulose has low compatibility in extreme wastewater, such as extremely acidic or extremely alkaline conditions, resulting in shorter lifespans [20]. A ceramic membrane based on γ-Al_2_O_3_ or SiC with a good hydrophilic surface proved to be a promising solution for oil/water separation; however, ceramic membranes have at least three limiting factors: (a) brittle mechanical strength; (b) high-pressure pulse backwashing consumes a lot of energy and results in easy break up after a certain operation cycle, and as such, many researchers and applications prefer material flexibility [21,22]; and (c) membrane pore size can be increased after a certain time operation under huge pressure scouring, which limits its rejection capability [23].

Very recently, inspired by the antiwetting properties of oil droplets on fish scales, a new idea of underwater superoleophobic surfaces was proposed [24]. Following this concept, many superhydrophilic in air and superoleophobic in water surfaces have been achieved in various ways [25,26]. Among them, two main approaches have been applied to construct antiwetting surfaces in water. One is surface fictionalization by hydrogel coating. This strategy is a typical and feasible approach for fabricating hydrophilic membranes. For example, Feng et al. [27] fabricated a hydrogel coated mesh with underwater superoleophobicity to separate the immiscible oil/water mixture. Liu et al. [28] coated inorganic oxides such as cooper oxide to construct a low adhesive superoleophobic surface. However, the coated membranes have obvious drawbacks of the weakened or unstable hydrophilic property under harsh conditions [17,29]. The other popular method is grafting nano-structures on metal mesh. For example, Zhang et al. [30] assembled TiO_2_ nanoparticles on the surface of stainless steel mesh separating water from an oil/water mixture effectively. For a further step, Zhang and co-workers also fabricated a nanowire-haired Cu(OH)_2_ on a copper mesh, and this resulting membrane was capable of separating oil/water mixtures with high efficiency under alkaline conditions [31]. However, these regenerated membranes on metal mesh have many disadvantages such as large pore size and poor flexibility, weakening the applications in oil/water separation [32].

Therefore, the development of a highly efficient, flexible membrane with long-term usability, low membrane fouling and feasible fabrication process is greatly desired. The core–shell structured microfiltration membrane with hydrophilic surface was reported in our previous study and the resulting membrane shows good microfiltration performance [33]. In the present study, we aim to (1) the detailed fabrication process of an organic/inorganic superflexible and superhydrophilic nanofiber membrane; (2) the properties (wettability, flexibility, mechanical property) of the resulting membrane; (3) the oil/water separation performance of the resulting membrane; and (4) tentative conclusions of formation mechanisms of the resulting membrane.

## 2. Experimental Section

### 2.1. Materials

Poly(vinylidene fluoride-co-hexa-fluoropropylene) (PVDF-HFP, *M*w = 400,000) and cupric acetate monohydrate were supplied by Aladdin Industrial Corporation, Shanghai, China. *N*,*N*-dimethyl formamide (DMF), ammonia water, and acetone were all purchased from Sinopharm Chemical Reagent Co., Ltd. (Suzhou, China). All reagents were used as obtained without further treatment.

### 2.2. PVDF-HFP/Cu(CH_3_COO)_2_ Nanofibrous Membrane Prepared by Electrospinning Process

PVDF-HFP (11 wt %) was dissolved in binary solvent of DMF/acetone (weight ratio of 5:5) at room temperature for 4 h. Then, the cupric acetate was added into the as-prepared solutions with different weight ratios of cupric acetate monohydrate/PVDF-HFP (1:2, 1:3, 1:6). Then the mixed solutions were stirred for 20 min under 70 °C to achieve blue solutions. During the electrospinning process, the collector distance was kept at 25 cm and a voltage of 25 kV was applied. The ambient relative humidity (RH) and temperature used in the spinning process were 50 ± 2% and 25 ± 2 °C, respectively. Lastly, the PVDF-HFP/Cu(CH_3_COO)_2_ nanofibrous membrane was fabricated and named as P/Cu(CH_3_COO)_2_ membrane.

### 2.3. PVDF-HFP/CuO Nanofibrous Membrane Prepared by Heating Process

The as-prepared P/Cu(CH_3_COO)_2_ membrane was put into an electronic oven under 120 °C for 24 h. The heating process was carried out to convert Cu(CH_3_COO)_2_ into CuO, acting as the seed for CuO nanosheet nucleation and crystallization in the following steps. After reaction, the PVDF-HFP/CuO nanofibrous membranes were fabricated and named as P/CuO membrane.

### 2.4. PVDF-HFP/CuO-Nanosheet Nanofibrous Membrane Prepared by Hydrothermal Process

The as-synthesized membranes after the heating process were immersed in the prepared growth media (Figure S5). The heat-resistant glass bottles were used as hydrothermal reactors to hold the growth media and the P/CuO membrane. Hydrothermal reactions occurred in an electronic oven under different temperature (40, 60, and 80 °C, respectively). The resulting nanofibrous membranes were cleaned by deionized water and then dried in oven at 40 °C for 5 h to remove the residual solvent. Thereafter, the membranes equilibrated at room conditions in air for several days before the water contact angle (CA) measurements. After the hydrothermal process, the PVDF-HFP/CuO-nanosheet nanofibrous membrane was produced and named as P/CuO-nanosheet membrane.

### 2.5. Characterizations

The resulting membrane morphology was observed by an FE-SEM (JEOL 7600F, Akishima, Japan) at 20 °C, 65 RH. The fiber diameters were calculated by measuring at least 100 fibers at random using the Image J program. Fourier transform infrared spectroscopy (FTIR, Nicolet 5700, Waltham, MA, USA) and X-ray diffraction (XRD, X’ Pert-pro MRD, Philips, Almelo, The Netherlands) were used to study the structure of the resulting nanofibrous membranes. In the testing process, the XRD scan rate was set as 4°/min with the 2θ ranging from 10 to 80°, and the FTIR spectrum ranged from 400 to 4000 cm^−1^. The FTIR and XRD results were corrected by the Savitzky–Golay method with the points of window of 20 and the polynomial order of 1.

The wetting abilities of the resulting membranes were characterized by an optical CA meter system (Krüss DSA100, Krüss, Hamburg, Germany). For underwater oil CA measurement, the membrane was firstly immersed in water, then a trichloromethane droplet (6 μL) was dropped onto the membrane. The final values were the average of five droplets based on different locations. An Olympus TH4-200 microscope was used to capture the optical microscopy images before and after separation.

A thermogravimetric analyzer (TGA (Symonston, Australia), TGA-4000, PerkinElmer, Waltham, MA, USA) was used to examine the water content in the filtrated product. In this process, the sample was heated from room temperature to 110 °C at a rate of 5 °C/min and held for 60 min. The separation efficiency was calculated by the following equation:

$$R=(1-W_{2}/W_{1})\times 100\%$$

where, the *R* is the rejection rate, *W*_2_ is the oil percentage after separation, and *W*_1_ is the oil percentage before separation.

The size distribution of oil droplets in the mixture was measured by a laser particle analyzer (Mastersizer-2000, Malvern, UK). The oil–water mixtures were prepared by mixing *n*-hexane, olive oil, cooking oil, and lubricant oil with water in a weight ratio of 1:10, respectively. The nitrogen provided the pressure (0.3 bar in the present study). During the separation experiment, the mixtures were poured into the filtration cup and separated by the membrane at the cup bottom. Then the flux was obtained by the following equation:

$$J=\frac{V}{t\times s}$$

where *J* is the flux (Lm^−2^ h^−1^), *V* is the mixture volume, *t* is the time, and *s* is the resulting membrane area in the separation experiment.

## 3. Results and Discussion

### 3.1. Evolution of P/CuO-Nanosheet Membrane Formation Process

**Figure 1:**
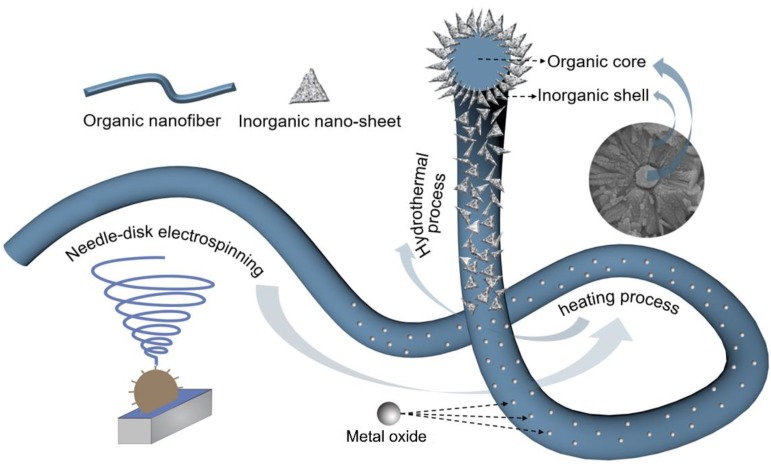
Schematic diagram of the evolution of the P/CuO-nanosheet membrane.

In this study, we introduced a facile process including an in-situ growth method to construct a delicate core–shell structured nanofibrous membrane. To vividly explain this process, a schematic diagram was drawn to explain the membrane formation evolution, as seen in Figure 1. Clearly, this integrated facile approach covers three major steps with different functions. Step 1 is continuous spinning of the PVDF-HFP nanofiber membrane by needle-disk electrospinning. Nanofiber high throughput is crucial to practical application [34]. Therefore, needle-disk electrospinning, which has demonstrated fabricating nanofiber with high quality and high throughput under a lower applied voltage, was applied to fabricate the nanofibrous membrane (Figure S1, Supplementary Information (SI)) [35,36]. Here, the modified electrospinning method was applied to aim at high throughput of the as-prepared membranes with greater uniformity compared to the conventional single-needle spinning process. After this procedure, P/Cu(CH_3_COO)_2_ membrane was fabricated (Figure 1). In Step 2, the heating process is helpful to form CuO nanoseeds on PVDF-HFP nanofibers, which is the crucial step for the formation of CuO nanoseeds to facilitate the nucleation and crystallization of CuO nanosheets [37]. The as-prepared membrane was heated in an oven at a temperature, inducing a cupric acetate reaction. After the reaction, the cupric acetate turned to cooper oxide and spread on the surface of the PVDF-HFP nanofiber (Figure 1). In Step 3, a low temperature hydrothermal reaction is intended to graft/anchor CuO nanosheets on PVDF fibers for stable applications. Previous reports of hydrothermal temperature were higher than 100 °C, even up to hundreds of degrees, which is not economical for scale up production owing to high energy consumption [38,39]. By contrast, the hydrothermal temperature in this study was as low as 60 °C, which was an energy saving approach and easy for scale up production. In this hydrothermal process, CuO nanosheets first nucleate on the well-formed nanoseeds and then crystallize into nanosheets with specific crystal properties. Rather than a physical mixing process, the chemical reaction initiates the nucleation, and crystal formation of CuO nanosheets enhances the anchoring affinity between CuO and PVDF-HFP nanofibers, which leads to excellent mechanical strength properties of the resultant membrane (Figure 1).

### 3.2. The Morphology and Structure of the Resulting Membranes

**Figure 2:**
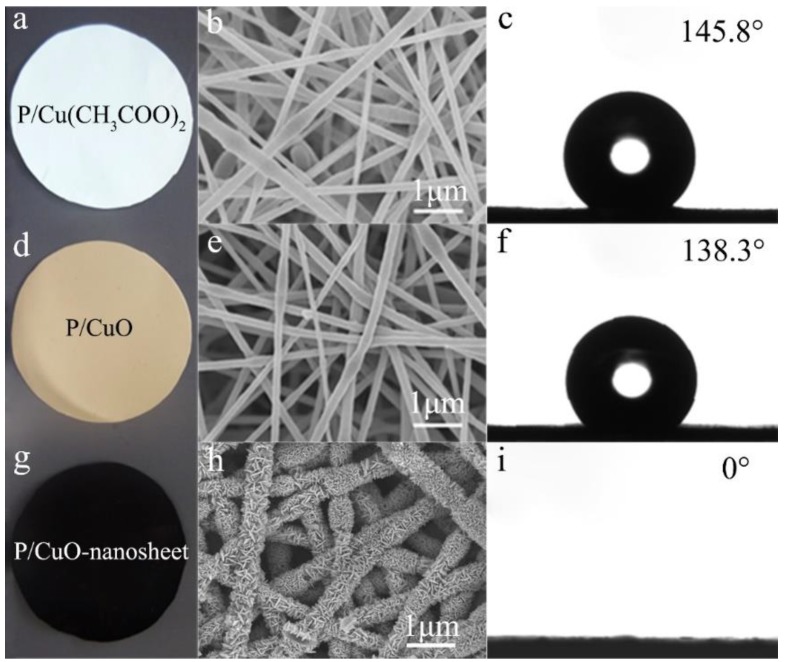
The optical image of (**a**) P/Cu(CH_3_COO)_2_, (**d**) P/CuO, and (**g**) P/CuO-nanosheet membranes; the morphology of (**b**) P/Cu(CH_3_COO)_2_, (**e**) P/CuO, and (**h**) P/CuO-nanosheet membranes; the wettability of (**c**) P/Cu(CH_3_COO)_2_, (**f**) P/CuO, and (**i**) P/CuO-nanosheet membranes.

**Figure 3:**
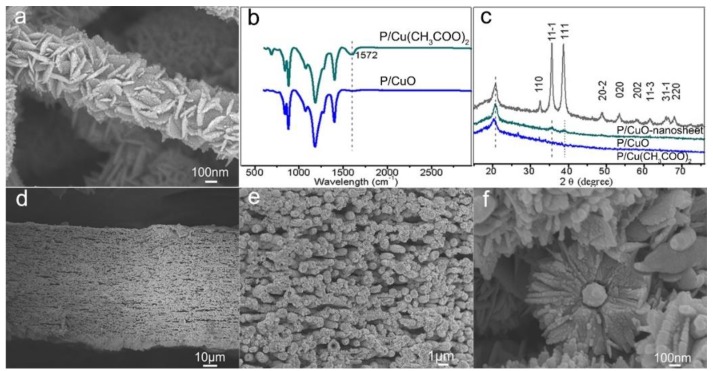
Morphology and structure of core–shell P/CuO-nanosheet membrane: (**a**) the morphology amplified by FE-SEM; (**b**) the FTIR spectrum of P/Cu(CH_3_COO)_2_ and P/CuO membrane; (**c**) the XRD spectrum of P/Cu(CH_3_COO)_2_, P/CuO, and P/CuO-nanosheet membranes; (**d**–**f**), the cross-section morphology with different magnification (**d**) ×700, (**e**) ×5000, and (**f**) ×60,000.

The morphology of the resultant membranes after each step are present in Figure 2. Obviously, the hydrophilicity property was enhanced gradually after heating (burning off organic residuals and forming CuO nanoseeds) and hydrothermal process (forming CuO nanosheet crystals). As shown in Figure 2a, the color of as-spun membrane was light blue, which was attributed to the cupric acetate. The resulting PVDF-HFP/cupric acetate nanofibers showed a smooth surface (Figure 2b) with fiber diameter of 198 ± 24 nm and narrow diameter distribution from 140 nm to 280 nm (Figure S2). Due to the PVDF-HFP hydrophobicity, the resulting composite membrane showed good hydrophobic properties with water CA 145.8°. After the heating process, the color changed to brown (Figure 2d), and the fiber diameter decreased to 161 ± 21 nm (Figure S2) owing to fiber shrinking and remodeling after residual solvent evaporation under heating. Meanwhile, the water CA decreased to 138.3° (Figure 2f). Obviously, the changed color and decreased water CA indicated the formation of CuO on the PVDF-HFP surface after the heat reaction. Finally, the color changed to black, and a few nanometers in thickness and 100–300 nm in length nanosheet structure was achieved (Figure 2h and Figure 3a), suggesting the growth of the CuO nanosheet after hydrothermal process. Moreover, the wettability of the resulting membrane changed dramatically from 138.3° to 0° (Figure 2i), which was as a result of the formation of a CuO nanosheet on the PVDF-HFP membrane. In order to reveal the effect of hydrothermal temperature on the resultant membrane, a systematic study with various P/CuO membranes was synthesized under different temperatures, with results showing in Figure S3, so as to determine the optimal hydrothermal reaction temperature. The experimental results demonstrated that the weight ratio of PVDF-HFP/cupric acetate and hydrothermal temperature had a great impact on the membrane morphology. With the decrease of the cupric acetate ratio, nanosheets decreased on the nanofiber surface, and no apparent nanosheets were observed when the weight ratio of cupric acetate/PVDF-HFP was 1:6 (Figure S3). A relatively high temperature of 40 °C did not lead to obvious membrane morphology (Figure S4). However, further temperature increases up to 80 °C resulted in tight nanosheets growing on the fiber surface because of the increased growth rate of nanosheets under 80 °C (Figure S4). Therefore, a growth temperature of 60 °C presented a mild growing process (Figure S4).

The cross-section morphology demonstrates the core–shell structure. As shown in Figure 3d, the membrane thickness was about 90 μm. It can be seen from Figure 3e that nanosheets covered all the surface of PVDF-HFP nanofibers, constructing a PVDF-HFP core and nanosheet shell. The amplified Figure 3f further demonstrates the core–shell structure. The brittle fracture interface under nitrogen conditions shows the inner PVDF-HFP nanofiber and external nanosheet (Figure 3f). This delicate structure integrates the advantages of the organic polymer with the inorganic nanosheet. The FTIR spectrum indicated that the –COO– radical existing in the P/Cu(CH_3_COO)_2_ membrane reacted after the heating process, as seen by the disappearance of 1572 cm^−1^ after the heating process (Figure 3b) [40]. Taking all the above factors like color, wettability change, and the FTIR spectrum change after the heating process into account, it was concluded that cupric acetate reacted and transformed into copper oxide. We speculated that cupric acetate reacted with water in the oven and changed to CuO and CH_3_COOH. Then, the CH_3_COOH volatilized at 120 °C, leaving CuO nanoseeds spreading on the surface of PVDF-HFP nanofibers (Figure 3).

Cu(CH_3_COO)_2_ + H_2_O → CuO + 2CH_3_COOH↑

The XRD pattern further confirmed the growth of CuO on PVDF-HFP nanofiber surface. As shown in Figure 3c, after the heating process, the two peaks at 35.7° and 38.8° corresponded to the characteristic peak of CuO [41]. What is more, after the hydrothermal process, the peaks at 32.6°, 35.7°, 38.8°, 48.9°, 53.5°, 58.1°, 61.7°, 66.1°, and 68.1° indicated the monoclinic system CuO crystals, which agreed with the values in the standard card (JCPDS) [42,43]. Moreover, the extremely strong peaks suggested high crystallinity of the CuO nanosheet on the PVDF-HFP backbone (Figure 3c).

### 3.3. The Wettability and Mechanical Properties of the P/CuO Nano-Sheet Membrane

**Figure 4:**
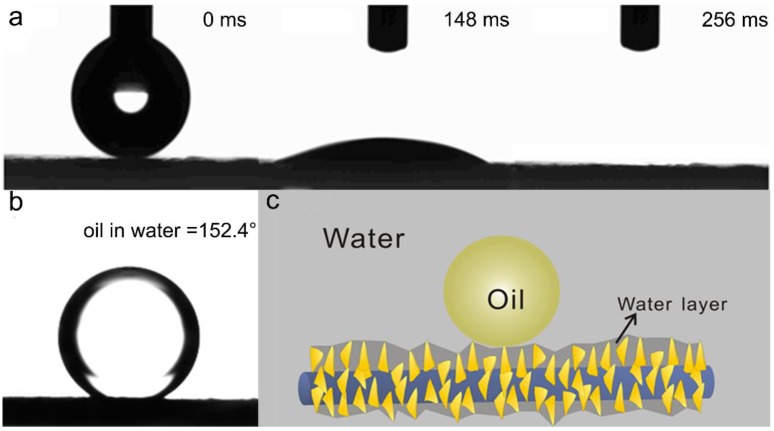
The wettability of the resulting P/CuO-nanosheet membrane: (**a**) the dynamic process of water droplets spreading on the membrane; (**b**) the underwater oil CA of the membrane; (**c**) the schematic diagram of the proposed water layer between the oil/water/solid interfaces.

The extremely rough CuO nanosheet on the fiber surface endows the membrane special wettability. As shown in Figure 4a, when a water droplet contacted the membrane surface, the droplet spread quickly and a zero water CA was achieved. Interestingly, the entire process was completed within 256 ms, indicating superior water wetting properties of the membrane (Figure 4a). The outstanding wettability in air always leads to a good underwater oil anti-wetting property [32], which, as confirmed by Figure 4b, the underwater oil CA reached 152.4°. The superhydrophilic and underwater superoleophobic properties were attributed to the extra rough structure resulting from CuO nanosheets on the membrane and the hydrophilic nature of CuO. When the nanosheet membrane was immersed in water, water was immediately trapped into the CuO nanosheet structures, forming an oil/water/solid interface in the presence of oil (Figure 4c). In other words, the trapped water acted as a repulsive layer for oils to make contact with the membrane directly. Owing to such surface properties, the resulting membrane allowed only the water to pass through and suggested a potential application in oil/water separation.

**Figure 5:**
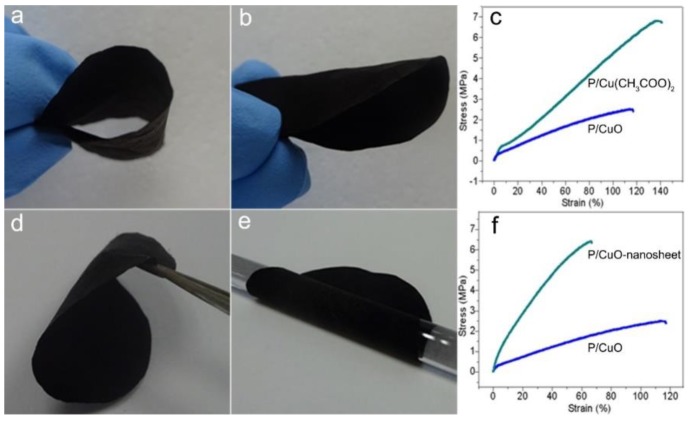
The optical image of P/CuO-nanosheet membrane with alterable shape (**a**,**b**,**d**,**e**); (**c**,**f**), the mechanical properties of P/Cu(CH_3_COO)_2_, P/CuO membranes, and P/CuO-nanosheet membranes.

Previous metal-based membranes always have poor flexible though they had good underwater oleophobicity. The membranes, in our study, possessed a polymer core and an inorganic shell, making it excellent in terms of flexibility as well as good underwater oleophobicity. As presented in Figure 5a,b,d,e, the resulting membrane could be folded for various desired shapes. The supporting video further confirmed the high flexibility of the membrane by blowing the membrane with a hair dryer. The P/Cu(CH_3_COO)_2_ membrane showed good mechanical properties with the stress at break 6.77 MPa and elongation at break 135.79%. However, the heating process weakened the mechanical properties, resulting in the stress at break 2.49 MPa and elongation at break 115.68% for the P/CuO membrane. Interesting, the stress at break of P/CuO-nanosheet membrane increased to 6.44 MPa and elongation at break decreased to 66.23%. It can be speculated that the formed CuO-nanosheet enhanced the linkage force between the fibers, favoring the increase of stress at break and the decrease of elongation at break.

In addition, the membrane held good stress at break, sacrificing with the strain at break after the whole process (Figure 5c,f). Therefore, the super flexibility accompanied by an actable mechanical property broadens the membrane for practical industrial applications.

### 3.4. The Membrane Performance for Oil/Water Separation

**Figure 6:**
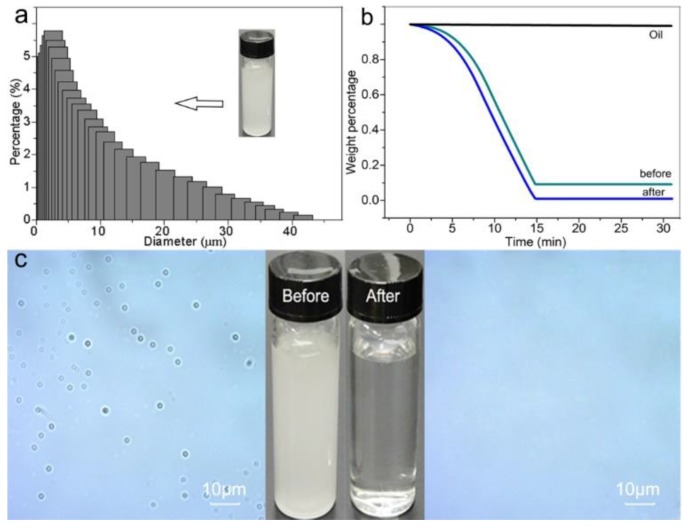
(**a**) the size distribution of oil droplets in mixture (olive oil); (**b**) the TGA curve of olive oil (as comparison), before and after separation; (**c**) the macroscope image of the solution before and after separation.

For the oil/water separation process, the oil/water mixtures were separated by a home-made setup (Figure S6) with the results being illustrated in Figure 6. As shown in Figure 6a, the model oil/water mixture (olive oil) showed the size distribution of oil droplets from 1 to 40 μm, while previous metal-based membranes could not be separated with a high rejection rate due to the large membrane pore size [44]. The size distribution of oil droplets was further characterized by the microscopy images, which were in accordance with the particle analyzer measurement results (Figure 6c, left). After separation, a clear filtrate was collected (Figure 6c, right) with a high separation efficiency higher than 99.8% (Figure 6b), highlighting that the membrane was effective in separating oil/water mixtures with oil droplets sizes large than 1 μm.

**Figure 7:**
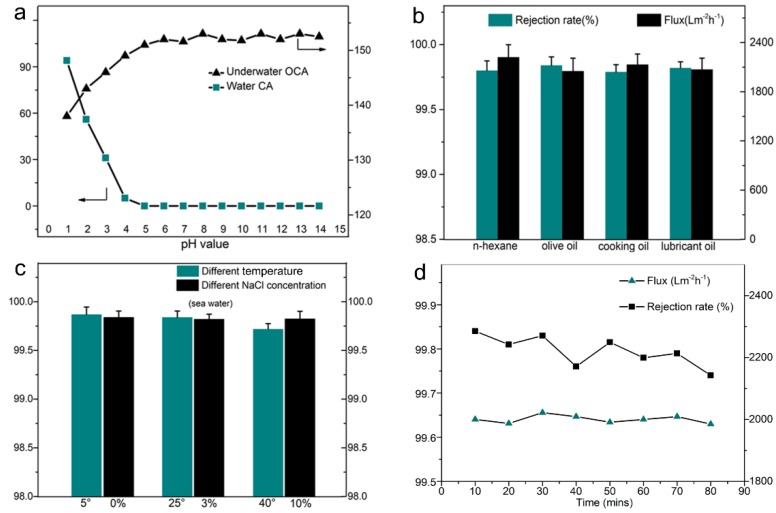
The oil/water mixture separation process: (**a**) the water CA and underwater oil CA under different pH value conditions; (**b**) the rejection rate and flux of different oil/water mixtures; (**c**) the rejection rate under different penetrating temperature and NaCl concentration (olive oil as the model oil); (**d**) the rejection rate and flux under 80 min (olive oil as the model oil).

Furthermore, the membrane practical application ability was evaluated by pH stability, separation of various mixtures, separation at different temperatures/ionic concentrations, and long-term usability. Figure 7a shows the membrane CA and underwater oil CA after immersing in different pH solutions for 24 h. As illustrated in Figure 7a, the CA increased, and underwater oil CA decreased dramatically with pH value less than 4.0, indicating the membrane poor stability properties in strong acid conditions. However, the membranes held the superhydrophilic and underwater superoleophobic properties with pH values larger than 5.0, suggesting good stability against alkaline conditions. The rejection rate for olive oil, cooking oil, and lubricant oil were all higher than 99.8% with acceptance flux higher than 2050 Lm^−2^ h^−1^ (Figure 7b). As shown in Figure 7c, the mixture temperature affected slightly the separation efficiency due to the change in oil-in-water solubility with temperature increases [45]. Nevertheless, the separation efficiency was still higher than 99.7%, indicating that the membrane could be used in a broad temperature range. In the practical separation process, ions always existed in filtrate. Figure 7c also shows that NaCl (model ion) concentrations had no significant influence on the membrane performance, demonstrated by the separation efficiency higher than 99.8%. The long-term usability is a key indicator for practical application. As shown in Figure 7d, the membrane could keep the flux and separation efficiency for 80 min without an obvious decrease for the model oil/water mixture (olive oil), suggesting its superior antifouling properties without sacrificing the product quality and productivity. This outcome was contributed to by the water layer formed between the oil and the membrane [46]. Thus, the membrane holds a good anti-fouling property and can be applied for long-term use. Ultimately, the results above suggest that the core–organic/inorganic membrane has great potential in practical oil/water separation.

### 3.5. The Proposed Formation Mechanism of P/CuO Nano-Sheet Membrane

**Figure 8:**
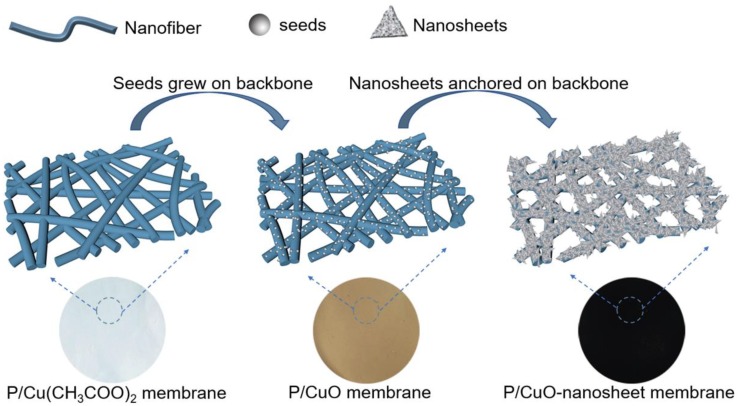
The schematic diagram of proposed formation mechanism for P/CuO nanosheet membrane.

Based on the morphology and structure characterization, the formation mechanism of core–shell P/CuO-nanosheet was proposed to give more insightful opinions for future researchers and applications: The polymer and inorganic salt dissolved together to fabricate a PVDF-HFP/Cu(CH_3_COO)_2_ composite nanofibrous backbone (Figure 8). After the heating process, the Cu(CH_3_COO)_2_ was transferred to CuO, which provided the sites where CuO grew. Then, the resulting P/CuO membrane was immersed into the CuO growth medium. The growth medium was cuprammonia, and a simple preparation process was carried out, as seen in Figure S5. With the temperature increase, the reaction went to the direction of producing Cu(OH)_2_. Subsequently, the Cu(NH_3_)_4_^2+^ dehydrating reaction formed the crystal nucleus of the CuO nanosheet at the interface between the nanofiber and the growth medium. After the formation of the crystal nucleus, the original granules had a random orientation at the early stage of nucleation growth. After that, the adjacent original granules rotated and shared the same crystallographic orientation driven by the thermal dynamic force, leading to the formation of the CuO nanosheet [47]. The reactions in the hydrothermal process are speculated as follows:

(Cu(NH_3_)_4_)^2+^ + 2OH^−^ → Cu(OH)_2_ + 4NH_3_

Cu(OH)_2_ → CuO

or

NH_3_ + H_2_O → NH^4+^ + OH^−^

2OH^−^ + Cu^2+^ → CuO + H_2_O

or

Cu^2+^ + OH^−^ → Cu(OH)_2_

Cu(OH)^2^ → CuO

These reactions may happen simultaneously, synergistically forming the CuO nanosheet on the PVDF-HFP nanofiber backbone.

## 4. Conclusions

We have successfully demonstrated a core–shell organic/inorganic composite membrane featuring superhydrophilic, underwater superoleophobic, anti-fouling, super-flexible, and alkaline resistance properties. As a result of these synergic integrated advantages, this delicate structure membrane shows excellent oil rejection rates under different filtration temperatures and ionic concentrations. Notably, the easily prepared membrane, using a continuous spinning technology plus low temperature hydrothermal process, exhibits high product quality by rejecting oils with high efficiency, high productivity without obvious fouling tendency at a relatively low pressure driven process even under various conditions, and a longer usability period. Therefore, it is of great significance to believe this strategy can become a general and alternative approach to fabricate functional core–shell structure membranes that shows potential applications in practical oil/water separation and other aspects.

## Supplementary Materials

The following are available online at https://www.mdpi.com/2073-4360/11/6/974/s1.
