# Supplementary material for: Efficient Oil/Water Separation Membrane Derived from Super-Flexible and Superhydrophilic Core–Shell Organic/Inorganic Nanofibrous Architectures
Source: Polymers (Basel). 2019 Jun 3;11(6):974. doi: 10.3390/polym11060974 (PMC6632045; doi:10.3390/polym11060974)
Supplement: Supplementary file 1 [file polymers-11-00974-s001.zip › polymers-512728-final si/Supplementary materials-edit-clean.doc]

**Efficient Oil/Water Separation Membrane Derived from Super-Flexible and Superhydrophilic Core–Shell Organic/Inorganic Nanofibrous Architectures**

Zhi Liu^a*^, DetaoQin^c^, JianghuiZhao^a^, Quan Feng^a^, Zhengtao Li^b^

Hongwei Bai^b*^, Darren DelaiSun^b*^

*^a^School of Textile and Garment, Anhui Polytechnic University, Beijing Road, Wuhu, 241000, China.*

*^b^School of Civil and Environmental Engineering, Nanyang Technological University, 639798, Singapore.*

*^c^Energy Research Institute @ NTU, Interdisciplinary Graduate School, Nanyang Technological University, 639798, Singapore*

*To whom correspondence should be addressed.

E-mail: liuzhi@ahpu.edu.cn; Tel.: 86-13485532640.

E-mail: baihw0001@gmail.com.

E-mail: ddsun@ntu.edu.sg; Tel.: 65-67906273; Fax: 65-67910676.

**Figure S1.** The schematic diagram of needle-disk electrospinning and traditional single needle electrospinning.

Compared with the traditional single-needle (hollow tube electrode) electrospinning, the needle-disk electrospinning uses the multiple solid needle as electrode with high-curvature, making the needle-disk electrospinning fabricated various nanofibers with high quality and high throughput under a lower applied voltage.

**Figure S2.** The diameter distribution of P/Cu(CH_3_COO)_2_ and P/CuO nanofibers.

**Figure S3.** The optical image and morphology of membranes fabricated under different weight ratio of PVDF-HFP/cupric acetate: a, 2:1; b, 3:1; and c, 6:1.

**Figure S4.** The membrane morphology with 1 h growth under a, 40 °C; b, 60 °C and c, 80 °C, respectively.

**Figure S5.** The optical image of copper acetate and cuprammonia solution.

The reactions in the preparation of cuprammonia (growth medium) are as follows:

Cu^2+^ + 2NH_3_.H_2_O = Cu(OH)_2_ + 2NH_3_^4+^
Cu(OH)_2_ + 4NH_3_.H_2_O = (Cu(NH_3_)_4_)^2+^ + 2OH^–^ + 4H_2_O

Cu^2+^ + 4NH_3_.H_2_O = (Cu(NH_3_)_4_)^2+^ + 4H_2_O

**Preparation of growth medium**

The cupric acetate was dissolved in deionized water at room temperature. Then, ammonia water was added drop by drop into the above solution. In this process, precipitation was formed at first and disappeared within a very short time. The color changed from transparent light blue to transparent navy blue, indicating the formation of cuprammonia.

**Figure S6.** The schematic diagram of home-made separation set up.
